# Integrated analysis of hydrothermal flow through pretreatment

**DOI:** 10.1186/1754-6834-5-49

**Published:** 2012-07-19

**Authors:** Veronique Archambault-Leger, Xiongjun Shao, Lee R Lynd

**Affiliations:** 1 Dartmouth College, Hanover, NH, 03755, USA; 2 DOE BioEnergy Science Center, Oak Ridge National Laboratory, Oak Ridge, TN, 37831, USA; 3 Mascoma Corporation, NH, 03766, Lebanon, USA

**Keywords:** Flowthrough pretreatment, Hydrothermal pretreatment, Biofuel, Cellulosic, Xylan, SSF, Consolidated bioprocessing, *Clostridium thermocellum*

## Abstract

**Background:**

The impact of hydrothermal flowthrough (FT) pretreatment severity on pretreatment and solubilization performance metrics was evaluated for three milled feedstocks (corn stover, bagasse, and poplar) and two conversion systems (simultaneous saccharification and fermentation using yeast and fungal cellulase, and fermentation by *Clostridium thermocellum*).

**Results:**

Compared to batch pretreatment, FT pretreatment consistently resulted in higher XMG recovery, higher removal of non-carbohydrate carbon and higher glucan solubilization by simultaneous saccharification and fermentation (SSF). XMG recovery was above 90% for FT pretreatment below 4.1 severity but decreased at higher severities, particularly for bagasse. Removal of non-carbohydrate carbon during FT pretreatment increased from 65% at low severity to 80% at high severity for corn stover, and from 40% to 70% for bagasse and poplar.

Solids obtained by FT pretreatment were amenable to high conversion for all of the feedstocks and conversion systems examined. The optimal time and temperature for FT pretreatment on poplar were found to be 16 min and 210°C. At these conditions, SSF glucan conversion was about 85%, 94% of the XMG was removed, and 62% of the non carbohydrate mass was solubilized. Solubilization of FT-pretreated poplar was compared for *C. thermocellum* fermentation (10% inoculum), and for yeast-fungal cellulase SSF (5% inoculum, cellulase loading of 5 and 10 FPU/g glucan supplemented with β-glucosidase at 15 and 30 U/g glucan). Under the conditions tested, which featured low solids concentration, *C. thermocellum* fermentation achieved faster rates and more complete conversion of FT-pretreated poplar than did SSF. Compared to SSF, solubilization by *C. thermocellum *was 30% higher after 4 days, and was over twice as fast on ball-milled FT-pretreated poplar*.*

**Conclusions:**

XMG removal trends were similar between feedstocks whereas glucan conversion trends were significantly different, suggesting that factors in addition to XMG removal impact amenability of glucan to enzymatic attack. Corn stover exhibited higher hydrolysis yields than bagasse or poplar, which could be due to higher removal of non-carbohydrate carbon. XMG in bagasse is more easily degraded than XMG in corn stover and poplar. Conversion of FT-pretreated substrates at low concentration was faster and more complete for C. *thermocellum* than for SSF.

## Background

Producing fuel from lignocellulosic biomass is of interest in light of pressing concerns about petroleum supply and climate change [1-4]. The main obstacle impeding production of cost-competitive cellulosic biofuels is the high cost of converting cellulosic feedstocks to reactive intermediates, termed biomass recalcitrance. In the case of biological conversion of cellulosic biomass to sugars, recalcitrance results from incomplete accessibility of attack by microbes and their saccharolytic enzymes due to structural features, heterogeneous composition, and chemical linkages between these components [5,6].

In the biomass conversion field, “pretreatment” refers to the process step that converts cellulosic biomass into a form amenable to biological attack. Various approaches to pretreatment allow hydrolysis yields of 90% or more, whereas low yields have been widely observed in the absence of pretreatment [7,8]. Pretreatment processes examined in the literature include exposure to acid or alkali, ammonia, lime, organic solvents, ionic liquids, and water, generally at elevated temperature and pressure [7-10]. Once cellulosic biomass is rendered amenable to biological attack, there are different approaches to ferment the substrate. In simultaneous saccharification and fermentation (SSF), cellulose hydrolysis and hexose fermentation occur in a separate unit operation from cellulase production. This configuration has several advantages, but the cost of cellulase remains a significant barrier [11-13]. In consolidated bioprocessing (CBP), cellulase production, cellulose hydrolysis, as well as hexose and pentose fermentations are all achieved in one process. CBP is in principle attractive because of streamlined processing and no costs for added enzymes, however development of requisite microorganisms is a work in progress.

Pretreatment has multiple objectives that are challenging to achieve at once. In particular, high cellulose reactivity is fostered by reaction at high temperature and long reaction times, yet such conditions commonly result in degradation of sugars and production of fermentation inhibitors. Addition of chemicals allows reactive solids to be obtained at lower temperatures and shorter times than would otherwise be possible, but involves costs due to purchase and/or recycle of the chemicals. Pretreatment accounts for a substantial fraction of the cost of processing biomass [14-16], has pervasive impacts on the performance and thus cost of hydrolysis and fermentation [9,14,17], and improvements in pretreatment are widely recognized as a key route to improving the cost-competitiveness of biomass conversion [10,18].

In addition to liquid phase composition, temperature and residence time, the configuration of pretreatment processes is also an important factor impacting performance. In particular, operation of pretreatment in a flow through (FT) configuration has been proposed and investigated to some degree, generally with a water or dilute acid liquid phase [14,19-22]. In FT pretreatment, the ratio of liquid and solid residence times, R_L/S_, is less than one, whereas in the absence of flow through – whether the process is operated in batch or continuous mode - R_L/S_ is equal to unity. As a result of liquid being removed from the reactor, solubilized sugars have less time to degrade, and recondensation of solubilized lignin and xylan on cellulose fibers upon cooling occurs to a lesser extent [14,21]. Consistent with this understanding, FT pretreatment typically achieves higher solids reactivity, higher xylan removal, less sugar degradation and substantially higher removal of lignin and other non-carbohydrate carbon compared to pretreatment in non flow through configurations at the same temperature and residence time [5,7-10,14]. The relationship between lignin removal and xylan removal is nearly linear, and it has been suggested that lignin and xylan are removed as complexes and that lignin disruption is a key determinant of solids digestibility [14,22]. Other studies have looked at the mass ratio of liquid to solids [23,24] or flow velocity [19,20,25], indicating that fluid flow has an impact on pretreatment mechanism whereas dilution effects may not be significant below 10 w/v %. To realize the advantages of FT pretreatment in a practical context, it is necessary to address the mechanical complexities of arranging a bed of biomass for flow through configuration at scale while also avoiding unacceptably high energy requirements and sugar dilution.

While we are optimistic that such realization is possible, this will require integrated understanding of fluid mechanics, kinetics, heat and mass transfer, and how these are impacted by feedstock properties, operating conditions, and the choice of conversion system. In order to provide a foundation for such studies, we undertake here to evaluate performance metrics for FT pretreatment as a function of time and temperature for corn stover, sugar cane bagasse and poplar, and also to compare conversion of FT-pretreated poplar by SSF and by *C. thermocellum*, a candidate CBP organism [11]. We are not aware of a prior study that has evaluated FT pretreatment for such a range of feedstocks and conversion systems.

## Results and discussion

### Effect of poplar moisture content

**Figure 1:**
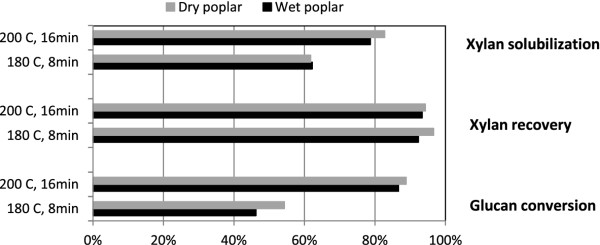
Comparison between dry and wet poplar pretreated at two different sets of time and temperature.

A control experiment was performed to study whether feedstock moisture impacted sugar recovery and enzymatic digestibility. Wet and dry milled poplar were FT pretreated at 180°C for 8 min (ratio of liquid and solid residence times, R_L/S_ = 0.25) and at 200°C for 16 min (R_L/S_ = 0.125), and xylan/mannan/galactan (XMG) recovery and solubilization along with glucan conversion after SSF were analyzed. SSF results were based on yeast fermentation (*Saccharomyces cerevisae* strain D5A) prepared in YPD medium supplemented with 16.7 mg cellulase/g glucan (10 FPU/g glucan) and 30 IU/g glucan Novozyme β-glucosidase. As shown in Figure 1, glucan conversion, XMG recovery and XMG solubilization appear to be higher for dried substrate than wet substrate, although the difference is not statistically significant. The standard error, estimated from the triplicate of a separate experiment, was 1.9%, 1.5% and 1.3% for glucan conversion, XMG recovery and XMG solubilization respectively. Since dry poplar sample is easier to mill and store, it was used for further analysis.

### Initial batch and FT pretreatments

**Figure 2:**
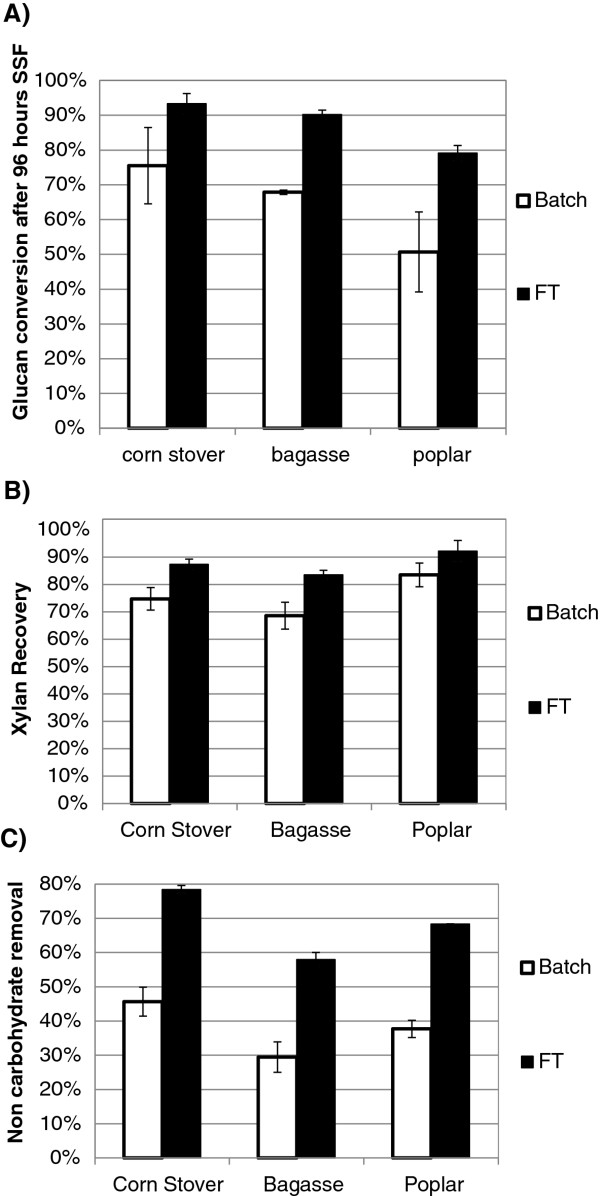
**Batch and FT pretreatment comparison for pretreated corn stover, poplar and bagasse. A**) glucan conversion, **B**) sugar recovery and **C**) extraction of non-carbohydrate carbon. Pretreatment Conditions: 220°C, 12 min (14 min for batch pretreatment), flow rate: 30 mL/min, particle size: 2 mm. Enzyme loading for SSF: 10 FPU/g glucan (11.7 mg enzymes/g solids). Initial substrate concentration: 20 g/L glucan. The error bars show one standard deviation on duplicates.

Milled corn stover, sugar cane bagasse and poplar were FT pretreated for 12 min at 220°C (R_L/S_ = 0.167) and batch pretreated for 14 min at 220°C (R_L/S_ = 1, 22.5 w/v %) to allow for the greater heat-up time in batch (see methods). After 96 h of SSF at the conditions specified above, glucan conversion was 93% for corn stover, 90% for bagasse, and 79% for poplar whereas batch pretreatment allowed about 75% glucan conversion for corn stover, 68% for bagasse and 50% for poplar (Figure 2A). Recovery of glucan and XMG fractions was evaluated based on the percent present in all forms (insoluble, oligomer, monomer) at the end of the experiment relative to that at the start of the experiment. On this basis, glucan recovery was 95-100% for batch and FT pretreatments on all substrates. XMG recovery ranged from 69% to 84% for batch pretreatment and from 84% to 92% for FT pretreatment, as shown in Figure 2B. Extraction of non-carbohydrate carbon (Figure 2), mostly lignin, was modest for batch (30% for bagasse, 38% for poplar and 46% for corn stover) but much more pronounced for FT pretreatment (58% for bagasse, 68% for poplar and 78% for corn stover). The higher solids reactivity, XMG recovery and removal of non carbohydrate carbon for FT pretreatment compared to batch observed in this study are consistent with results obtained by Liu and Wyman [20] and Yang and Wyman [14,23] for corn stover. This study showed that the higher solids reactivity, XMG recovery and removal of non carbohydrate carbon for FT pretreatment compared to batch were also observed with bagasse and poplar. For both FT and batch pretreatments, poplar’s conversion is lower and its XMG recovery higher compared with corn stover and bagasse. Moreover, the removal of non carbohydrate carbon is higher in corn stover than in the other substrates studied.

### Exploration of time and temperature for FT pretreatment

Data trends for milled poplar, bagasse and corn stover were visualized by plotting glucan conversion, removal of XMG and non-carbohydrate carbon, and XMG recovery as a function of severity, defined as log $Ro=log\left(t\frac{\exp^{T-100}}{14.75}\right)$[15], where t is the time of reaction (minutes) and T is the temperature of the reaction (°C). A range of severities from 3.2 to 5.1 was obtained by varying the time of pretreatment from 8 to 24 min (R_L/S_= 0.250-0.083) and the temperature from 180°C to 225°C.

#### Glucan conversion

**Figure 3:**
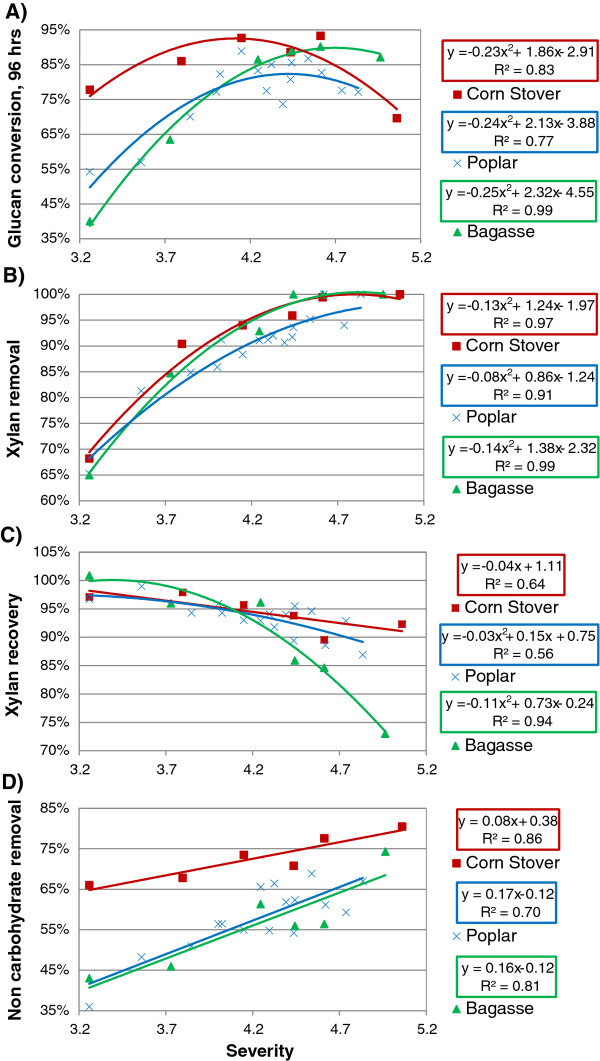
**Comparison between poplar, corn stover and bagasse.** Glucan conversion after 96 h (**A**), XMG removal (**B**), XMG recovery (**C**) and non-carbohydrate removal (**D**) against severity of pretreatment.

The conversion of glucan in pretreated biomass during SSF is a direct measure of the digestibility of the substrate. It was found, as illustrated in Figure 3A, that the conversion follows a second order polynomial fit with similar shape for corn stover, bagasse and poplar. The biomass is increasingly digestible as time and temperature increase until an optimum point is reached, beyond which glucan conversion decreases. The optimum point varies slightly between substrates, likely due to their differences in composition and structure. The optimal severity was 4.1 for corn stover (16 min, 200°C, R_L/S_= 0.125), 4.4 for poplar (16 min, 210°C, R_L/S_= 0.125), and 4.6 for bagasse (12 min, 220°C, R_L/S_= 0.167). The higher glucan conversion for corn stover at a lower severity is likely due to the higher non-carbohydrate removal compared to other substrates discussed below.

#### XMG removal/recovery

XMG removal followed similar trends for the different feedstocks tested, although slightly lower values were obtained for poplar, as illustrated in Figure 3B. Removal of XMG increased from 65% at a severity of 3.2 (8 min, 180°C, R_L/S_= 0.25) to 100% at a severity of 4.4–4.6 (16–24 min, 210°C, R_L/S_= 0.125–0.083). Since glucan conversion exhibits much more variability than XMG removal among the three feedstocks tested, factors other than XMG removal must affect the amenability to enzymatic attack. XMG recovery, shown in Figure 3C, is above 95% for all feedstocks at severities below 4.1 but decreases above a severity of 4.1 due to degradation. Degradation products data are provided in Additional file 1. The recovery decreases fastest for bagasse, indicating that XMG in bagasse is the most susceptible to degradation among the substrates tested due to chemical and morphological differences.

#### Extraction of non-carbohydrate carbon

**Figure 4:**
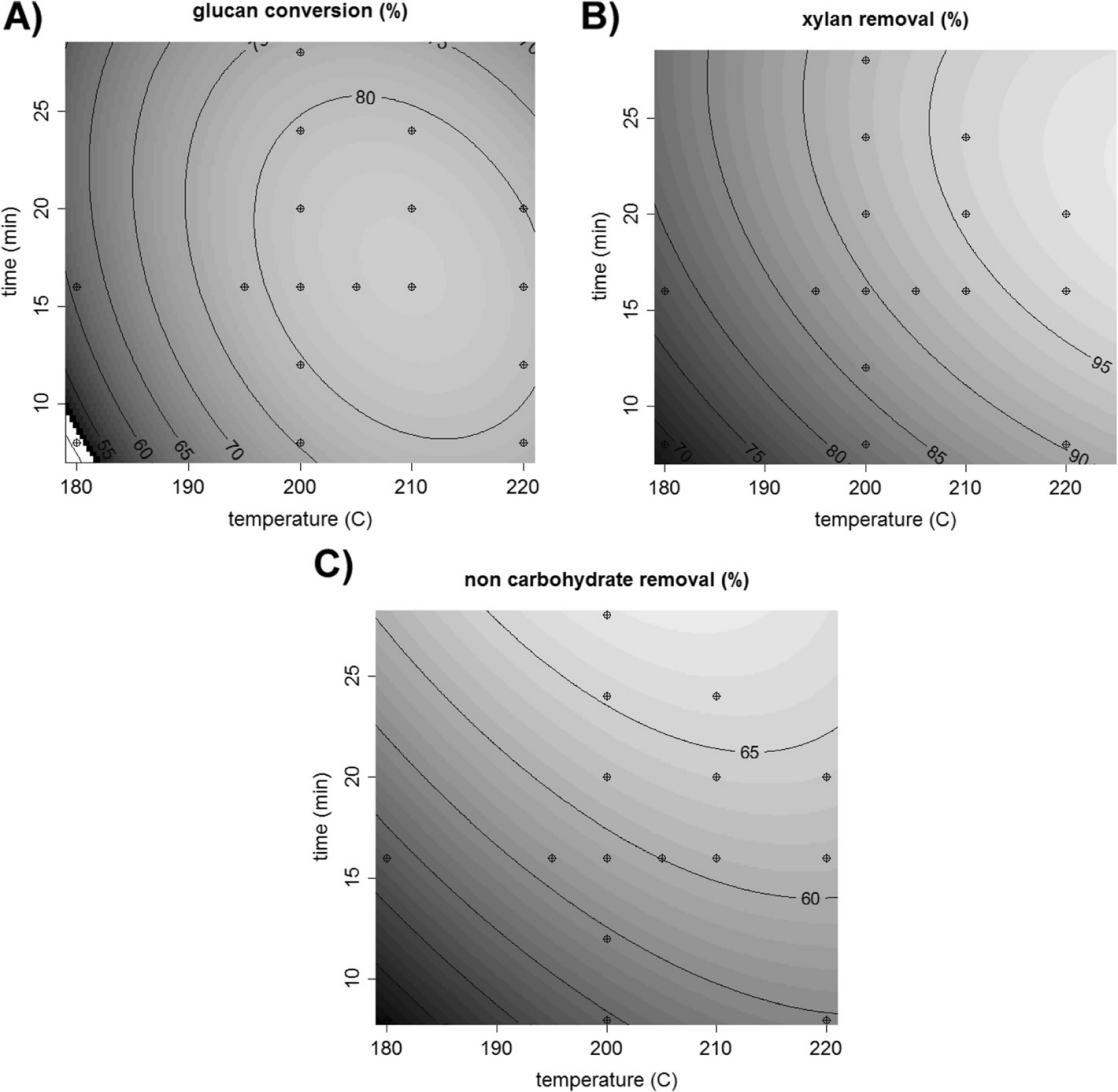
**Contour plots on poplaragainst time and temperature. A**) glucan conversion, **B**) XMG removal and **C**) non carbohydrate removal.

Removal of non-carbohydrate carbon was evaluated by evaluating the difference between total dry weight and carbohydrate content (anhydrous basis) after and before pretreatment. Removal of non-carbohydrate carbon, illustrated in Figure 3D, is not statistically different for poplar and bagasse, but is much higher for corn stover. It increases from about 40% to about 70% for poplar and bagasse and from 65% to 80% for corn stover at severities of 3.2 to about 4.9. This difference is a potential explanation for the higher amenability to SSF of corn stover pretreated at severities below 4.7 (Figure 1a and 4a), since lignin has been shown to impede glucan conversion [14,22].

### Optimization of FT pretreatment conditions on poplar

Central composite design was used to generate response surfaces for various performance metrics as a function of temperature and reaction time for milled poplar. Glucan conversion, XMG removal, and non-carbohydrate removal were evaluated. The response surfaces, shown in Figure 4, were fit to a quadratic model. The adjusted coefficient of determination (adjusted R^2^) and the p-value for the F-tests were used to evaluate the validity of the model and are provided in Additional file 2: Table S1 in supplemental materials. Additional file 2: Table S1 also presents the parameters of the quadratic model equations. All the models presented in this paper have p-values much smaller than 0.0003, indicating the models are statistically significant. The adjusted R^2^ values range from 0.76 to 0.95. The points marked on the contour plots represent data points (Additional file 3: Table S2 in supplemental materials).

As shown in Figure 4a, the model predicts that a maximum glucan solubilization of about 86% occurs at 210°C and 16 min (R_L/S_= 0.125). The XMG solubilized and recovered in the FT hydrolyzate is 5 to 15% lower than XMG removal, consistent with XMG degradation. Complete removal of XMG is beneficial for the fermentation of the pretreated solids, but degradation is not desired [5]. Thus, an optimal point was found at 24 min and 210°C (R_L/S_= 0.083) where all of the XMG is removed from the solid and 89% of the XMG was recovered in the hydrolyzate. A distinguishing feature of FT pretreatment is the high degree of solubilization of non-carbohydrate carbon. In particular, non-carbohydrate removal was 65% for pretreatment of 24 min or more at temperatures at or above 200°C.

### Exploration of conversion systems

Comparison of alternative conversion systems is of obvious interest, and has not been undertaken previously on milled FT-pretreated cellulosic feedstocks. C. *thermocellum* and SSF were compared on FT pretreated poplar (FTP), ball milled FT pretreated poplar (BMFTP), and Avicel, a laboratory microcrystalline cellulose.

**Figure 5:**
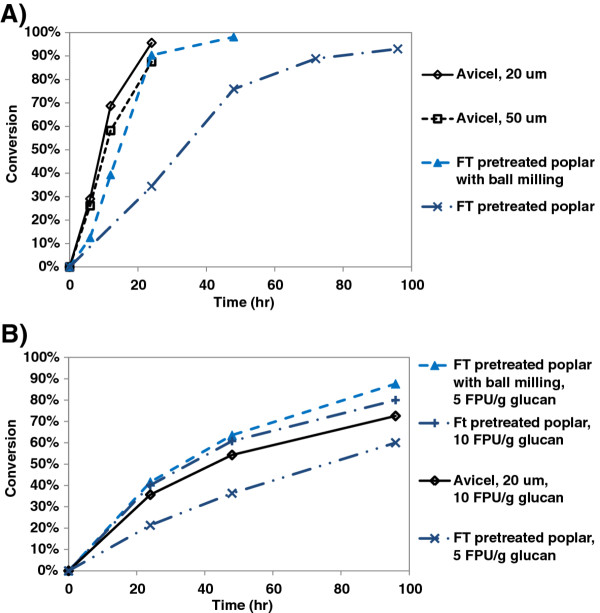
**Glucan conversion on various substrates for A) C.*thermocellum*and B) simultaneous saccharification and fermentation (SSF).**

Under the conditions tested, which are intended for intrinsic comparison and are not representative of an in-dustrial process, *C. thermocellum* solubilized all substrates tested more rapidly and more completely as compared to SSF (Figure 5). In particular, solubilization of FTP by C. *thermocellum* after 4 days (93%) was 32% higher than by SSF at 8.4 mg cellulase/g glucan (5 FPU/g glucan). Ball milling prior to hydrolysis substantially accelerated hydrolysis for both SSF and *C. thermocellum*, with *C. thermocellum* demonstrating higher rate and yield. In particular, *C. thermocellum* solubilized 98% of the cellulose in BMFTP in 2 days whereas SSF at 5 FPU/glucan required 5 days to achieve 88% solubilization.

## Conclusions

Hot water FT pretreatment provides highly digestible solids and high sugar recovery for various types of milled biomass. For example, 90% of the glucan in bagasse was converted after 96 h of SSF and 84% of the XMG was recovered when FT pretreated for 12 min at 220°C (R_L/S_= 0.167) versus 68% glucan conversion and 69% XMG recovery when batch pretreated for 14 min at the same temperature. It was found that the optimal reaction times and temperatures for FT pretreatment are 16 min at 200°C (R_L/S_= 0.125) for corn stover, 16 min at 210°C (R_L/S_= 0.125) for poplar and 12 min at 220°C (R_L/S_= 0.167) for bagasse. At those conditions, the glucan conversion after 96 h in SSF was 93% for corn stover, 86% for poplar and 90% for bagasse and the XMG recovery was 96% for corn stover, 97% for poplar and 85% for bagasse. Thus, corn stover gives high glucan conversion yields at substantially lower severity than poplar or bagasse. XMG removal is rather similar although perhaps a bit less for poplar. The fact that glucan conversion is more different than XMG removal suggests that factors in addition to XMG removal impact amenability of glucan to enzymatic attack. Higher removal of non-carbohydrate carbon was observed for corn stover than for poplar and bagasse, which may contribute to the lower severity required for corn stover pretreatment. XMG recovery is above 90% for all substrates below 210°C but is notably lower for bagasse at the high severities required to achieve high yields. C. *thermocellum* converts glucan more rapidly and completely than SSF under the conditions tested on Avicel and FT pretreated poplar. For example, after 4 days, C. *thermocellum* conversion of FT pretreated poplar was 32% higher than SSF.

## Methods

### Materials

Poplar (*Populus tremuloides*) obtained from Meriden, NH was harvested in the summer and 1 to 4” diameter trunks and branches were chipped (~ ½ inch largest dimension). The chips were allowed to dry to a moisture content of 7% in ambient air at room temperature. Wet poplar was never dried and its moisture content was measured to be 50%. Corn stover used for the CAFI project originally supplied by BioMassAgriProducts (BMAP, Harlan, IA) [8] was kindly provided by Dr. Bruce Dale’s lab at Michigan State University. Sugarcane from which bagasse was produced was harvested fresh in the winter from central Florida was provided by Mascoma Corporation (Lebanon, NH). All feedstocks were knife-milled (Model 3379 K35, Thomas Scientific, Swedesboro, NJ) to pass through a 2 mm screen. Bagasse was also sieved to remove dust and particles smaller than 105 μm. Flowthrough pretreated poplar was ball-milled for 60 min (Model no. SFM-3, MTI Corporation, Richmond, CA) when noted in the text. The composition of representative samples, shown in Table 1, was determined according to the NREL Laboratory Analytical Procedures (LAP’s) [24]. Avicel PH 105 was purchased from FMC Corporation (Philadelphia, PA). Spezyme CP cellulase was kindly provided by Genencor International Inc. (Rochester, NY) and Novozyme188 β- glucosidase was purchased from Sigma-Aldrich (St. Louis, MO). All samples were refrigerated at 4°C.

**Table 1:** Feedstock composition before pretreatment with the standard deviation on duplicates

**Feedstock**	**%glucan**	**%xylan**	**%arabinan**	**%lignin**
Corn Stover	33.2±1.6	23.2±0.4	2.7±0.04	17.2 [11]
Bagasse	40.4±1.3	23.6±0.6	1.5±0.1	20.9±1.9
Popular	37.8±0.5	16.1±1.3	0.9±0.2	21.9±0.5

### FT apparatus and experiments

The FT experiments followed a procedure similar to that described previously by Liu and Wyman [19,20]. The reactor was a 16 cm long stainless steel tube with an internal diameter of 2.1 cm, corresponding to a volume of 56 mL. Filter gaskets with 20 μm pore size, kindly provided by Mott Corporation (Farmington, CT), were used at the inlet and outlet of the tube reactor to contain the solids. All tubes and fittings were stainless steel 316 L purchased from Swagelok (Bangor, Maine). 12.6±0.5 g of feedstock was loaded in the reactor (22.5 w/v %) Water was pumped through the reactor using a Lab Alliance dual piston pump (Prep 100, Scientific Systems, PA) at 30 mL/min at room temperature to wet the solids. Once the outlet liquid was devoid of air bubbles, the heating coil and reactor were lowered into a fluidized sand bath controlled at the desired temperature. The start of the reaction time was set arbitrarily as the time when the reactor was lowered into the sand bath and the heating time was observed to be about 5 min by monitoring the temperature of the outlet water with a thermocouple. When the target reaction time was reached, the reactor was immersed in an ice water bath to quench the reaction. The water flow was stopped when the temperature at the outlet of the reactor dropped below 60°C. Pretreatment times were varied from 8 min to 28 min and reaction temperatures ranged from 180°C to 225°C. A triplicate was performed at 200°C for 12 min to estimate the error of replicate measurements.

### Batch experiments

The apparatus described above was also used for pretreatments without flow. The procedure was modified from most batch pretreatments reported in the literature [22]. The modified batch pretreatment was conducted with the same conditions as the FT pretreatment except that water flow was stopped once the wetting water was devoid of air bubbles. The heating time was experimentally observed to be about 7 min in separate experiments where a thermocouple was inserted in the center of the reactor. An extra two minutes was allowed for batch compared to FT pretreatment to ensure equivalent reaction times at the target temperature. For example, reaction time reported as 12 min means 12 min for FT and 14 min for batch. After a preset reaction time, the reactor immersed in an ice water bath for about 3 min until the reactor cooled to approximately 60°C and then the water flow was started again to collect the hydrolysate fraction of the pretreated mixture.

### Composition analysis

Compositional analysis of the solid and liquid fractions was determined using NREL Laboratory Analytical Procedures (LAP’s) [24]. Carbohydrates were analyzed via refractive index using an Aminex HPX-87 H column at 65°C on a Waters HPLC system (2695 Separations Module, Waters Corporation, Milford MA). Degradation products were analyzed via UV spectra using an Agilent Eclipse XD8-C18 column on a Thermo-Spectra System HPLC. Sugar recovery, XMG solubilization, XMG removal, non carbohydrate removal and glucan conversion (x) were calculated using equations 1, 2, 3, 4 and 5, respectively. The XMG solubilization in this paper corresponds to the XMG solubilized and recovered in the hydrolysate. Non carbohydrate removal is calculated using the concentration of carbohydrates in each solids sample (equation 4), which includes in this study glucose, xylose and arabinose. Mannose and galactose were measured once for each substrate and each was found to be less than one percent.

(1) $$sugar\;recovery(\%)=\frac{{\left(Sugar\;Weight\right)}_{\mathrm{hydrolyzate}}+{\left(Sugar\;Weight\right)}_{\mathrm{final}}}{{\left(Sugar\;Weight\right)}_{\mathrm{initial}}}\times 100$$

(2) $$XMG\;solubilisation\left(\%\right)=\frac{{\left(XMG\;Weight\right)}_{\mathrm{hydrolyzate}}}{{\left(XMG\;Weight\right)}_{\mathrm{initial}}}\times 100$$

(3) $$XMG(\%)=\frac{{\left(XMG\;Weight\right)}_{\mathrm{initial}}-{\left(XMG\;Weight\right)}_{\mathrm{final}}}{{\left(XMG\;Weight\right)}_{\mathrm{initial}}}\times 100$$

(4) $$non\;carbohydrate\;removal(\%)=\frac{\left(1-x_{carbohydrate\;carbon\text{,}\;initial}\right)*m_{total\;carbon\text{,}\;initial}-\left(1-x\right)*m_{total\;carbon\text{,}\;final}}{\left(1-x_{carbohydrate\;carbon\text{,}\;initial}\right)*m_{total\;carbon\text{,}\;initial}}\times 100$$

(5) $$x=\frac{{\left(Glucan\;Weight\right)}_{\mathrm{initial}}-{\left(Glucan\;Weight\right)}_{\mathrm{final}}}{{\left(Glucan\;Weight\right)}_{\mathrm{initial}}}$$

### SSF

SSF was carried out using a protocol similar to that described previously [25] with 20 g/L initial glucan loading for Figures 1, 2, 3, and 4 and 5 g/L initial glucan loading for Figure 5 for comparison with *C. thermocellum.* 5% (v/v) inoculation of *Saccharomyces cerevisae* strain D5A (NREL) was prepared via overnight culture for 16 h in YPD media (Sigma Y1375). The experiments were performed in 125 mL serum bottles (Bellco, Vineland, NJ), which were prefilled with the solid residue after pretreatment and media, sealed and purged with nitrogen. The bottles were sterilized by autoclaving at 121°C for 45 min and brought to room temperature prior to addition of enzymes and yeast. The Spezyme CP cellulase, assumed to contain 1 FPU per 0.6 mg of protein, was loaded at 5 or 10 FPU/g glucan, as noted in the text. It was supplemented by Novozyme188 β- glucosidase at an activity ratio of 3 IU per FPU. The medium used was developed by Kadam and Newman [26] and consists of 0.3% (v/v) corn steep liquor supplemented by 5 mM MgSO_4_. The inoculum was prepared from frozen stock in YPD media. The inoculated serum bottles were incubated at 37°C with 200 rpm shaking for the duration of the experiment. The remaining solids were processed and analyzed according to the composition analysis described above after 24 and 96 h.

#### *C. thermocellum* fermentation

*C*. *thermocellum* fermentation was carried out with 5 g/L initial glucan loading and 10% (v/v) inoculation of C. *thermocellum* ATCC 27405 (American Type Culture Collection, Manassas, VA). The experiments were performed in 125 mL serum bottles (Bellco, Vineland, NJ), which were prefilled with substrate and media, sealed and purged with nitrogen. The bottles were sterilized by autoclaving at 121°C for 45 min and then brought to room temperature before adding A, B, C, D, E and F solutions as described by Shao et al. [25]. The medium was prepared according to Zhang and Lynd [27] using chemically-defined media for thermophilic clostridia (MTC). The inoculum was prepared from frozen stock of a single isolated colony on 5 g/L Avicel PH 105 in MTC media. The inoculated serum bottles were placed in a 55°C shaking incubator (New Brunswick Scientific, Inova 4080) at 200 rpm for the duration of the experiment. Sample collection and processing were the same as described for SSF.

## Competing interests

Lee R. Lynd is a cofounder and share-holder of Mascoma Corporation, who may have a financial interest in the current study.

## Author’s contributions

VA performed experiments for optimizing pretreatment conditions and for comparing feedstocks, as well as drafted the paper. XS performed the experiments for comparing conversion systems. All authors participated in designing the study, have revised the paper critically for intellectual content and have read and approved the final manuscript.

## Supplementary Material

Additional file 1 **Figure S1.** Measured degradation products in the hydrolysate for FT corn stover, bagasse and poplar. Provides degradation products data explanatory for a decrease in sugar recovery. Click here for file

Additional file 2 **Table S1.** Quadratic models for FT pretreatment on poplar at various times and temperatures. Provides the mathematical model, the adjusted R^2^ value and the p-value for Figure 4. Click here for file

Additional file 3 **Table S2.** Compositional mass balances on pretreatment and SSF. Provides supporting data in grams for all Figures. Initial glucan is 1 g for Figures 1, 2, 3, and 4 and 0.25 g for Figure 5. SSF glucan residue is reported after 4 days. Click here for file
