# Supplementary figures and images for: Integrated analysis of hydrothermal flow through pretreatment
Source: Biotechnol Biofuels. 2012 Jul 19;5:49. doi: 10.1186/1754-6834-5-49 (PMC3495837; doi:10.1186/1754-6834-5-49)

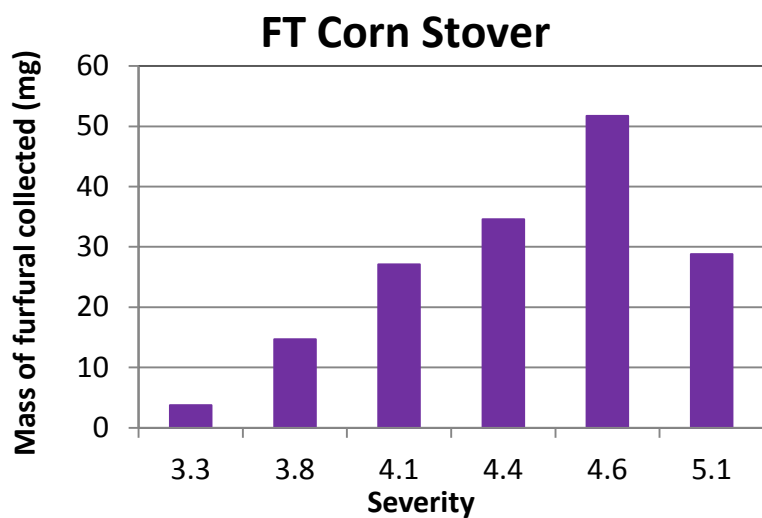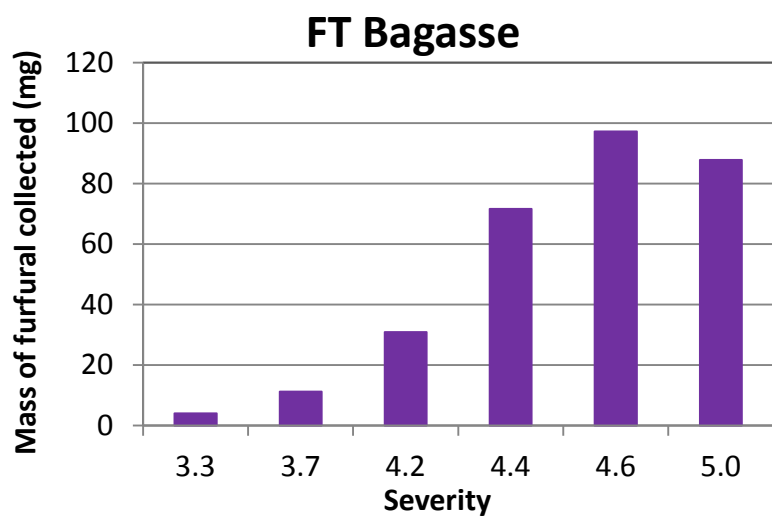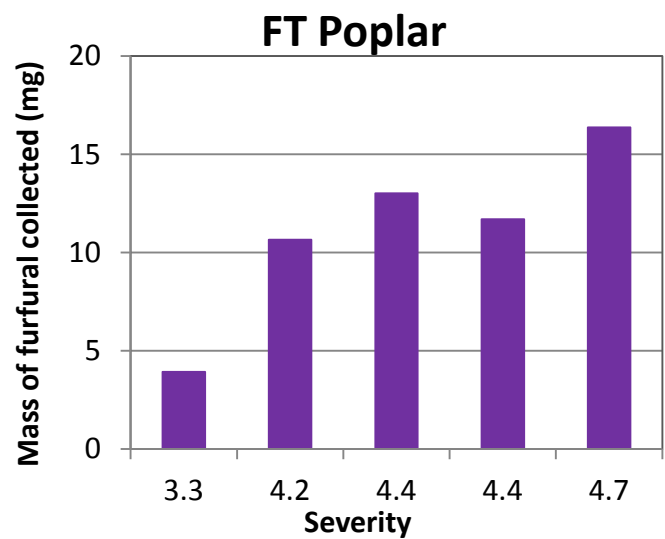

Supplement: Additional file 1 — Figure S1. Measured degradation products in the hydrolysate for FT corn stover, bagasse and poplar. Provides degradation products data explanatory for a decrease in sugar recovery. [file 1754-6834-5-49-S1.pdf]
