# Supplementary material for: Integrated analysis of hydrothermal flow through pretreatment
Source: Biotechnol Biofuels. 2012 Jul 19;5:49. doi: 10.1186/1754-6834-5-49 (PMC3495837; doi:10.1186/1754-6834-5-49)
Supplement: Additional file 2 — Table S1. Quadratic models for FT pretreatment on poplar at various times and temperatures. Provides the mathematical model, the adjusted R2 value and the p-value for Figure 4. [file 1754-6834-5-49-S2.pdf]

Table S1. Quadratic models for FT pretreatment on poplar at various times and temperatures.

	**Model**	**Adjusted R^2**	**p-value**
**Glucan conversion**	$-1,170 + 11.4\ T + 6.7\ t - 0.022\ t\ T - 0.0264\ T^2 - 0.061\ t^2$	0.76	0.00027
**Xylan solubilization**	$-820 + 8.4\ T + 0.46\ t + 0.0041\ t\ T - 0.0199\ T^2 - 0.030\ t^2$	0.95	3.0e-07
**Xylan removal**	$-290 + 3.0\ T + 3.3\ t - 0.0076\ t\ T - 0.0061\ T^2 - 0.035\ t^2$	0.89	2.8e-05
**Non-carbohydrate removal**	$-460 + 4.4\ T + 4.6\ t - 0.015\ t\ T - 0.0097\ T^2 - 0.019\ t^2$	0.83	0.00019
