# Supplementary material for: Integrated analysis of hydrothermal flow through pretreatment
Source: Biotechnol Biofuels. 2012 Jul 19;5:49. doi: 10.1186/1754-6834-5-49 (PMC3495837; doi:10.1186/1754-6834-5-49)
Supplement: Additional file 3 — Table S2. Compositional mass balances on pretreatment and SSF. Provides supporting data in grams for all Figures. Initial glucan is 1 g for Figures 1, 2, 3, and 4 and 0.25 g for Figure 5. SSF glucan residue is reported after 4 days. [file 1754-6834-5-49-S3.pdf]

Table S2. Compositional mass balances on pretreatment and SSF.

		time (min)	Temp (oC)	untreated glucan	untreated XMG	untreated arabinan	pretreated solids glucan	pretreated solids XMG	pretreated solids arabinan	hydrolyzate glucan	hydrolyzate XMG	hydrolyzate arabinan	SSF residue glucan
Figure 1	Wet poplar	8.00	180.00	3.90	1.66	0.09	4.08	0.50	0.00	0.21	1.03	0.09	0.54
		16.00	200.00	2.71	1.15	0.06	2.87	0.17	0.00	0.11	0.91	0.05	0.13
	Dry poplar	8.00	180.00	4.70	2.00	0.11	4.07	0.70	0.00	0.39	1.24	0.12	0.46
		16.00	200.00	4.70	2.00	0.11	4.13	0.23	0.00	0.41	1.66	0.12	0.11

			untreated glucan	untreated XMG	untreated arabinan	pretreated solids glucan	pretreated solids XMG	pretreated solids arabinan	hydrolyzate glucan	hydrolyzate XMG	hydrolyzate arabinan	SSF residue glucan
Figure 2	Batch	CS	3.92	2.87	0.33	3.50	0.67	0.04	0.25	1.56	0.24	0.24
			3.88	2.84	0.33	3.48	0.49	0.00	0.36	1.55	0.26	0.24
		Bagasse	4.40	2.66	0.16	3.95	0.39	0.00	0.25	1.34	0.11	0.32
			4.99	2.82	0.20	4.77	0.78	0.00	0.23	1.26	0.17	0.32
		Poplar	3.96	1.96	0.11	3.88	0.60	0.00	0.29	1.05	0.11	0.49
			4.84	1.88	0.07	4.73	0.27	0.00	0.04	1.21	0.06	0.49
	FT	CS	3.95	2.89	0.33	3.39	0.00	0.00	0.62	2.59	0.35	0.07
			3.88	2.84	0.34	3.50	0.00	0.00	0.12	2.45	0.28	0.07
		Bagasse	4.73	2.86	0.17	4.22	0.00	0.00	0.35	2.41	0.19	0.10
			4.87	2.82	0.14	4.45	0.09	0.00	0.30	2.21	0.14	0.10
		Poplar	4.72	2.01	0.12	4.11	0.12	0.00	0.44	1.79	0.12	0.21
			4.72	2.01	0.12	4.05	0.17	0.00	0.43	1.63	0.13	0.21

		time (min)	Temp (oC)	untreated glucan	untreated XMG	untreated arabinan	pretreated solids glucan	pretreated solids XMG	pretreated solids arabinan	hydrolyzate glucan	hydrolyzate XMG	hydrolyzate arabinan	SSF residue glucan
Figure 3	Corn Stover	8.00	180.00	4.30	3.00	0.35	2.95	0.95	0.04	0.36	1.96	0.28	0.22
		14.00	190.00	4.30	3.00	0.35	3.97	0.29	0.00	0.41	2.65	0.38	0.14
		16.00	200.00	4.30	3.00	0.35	3.93	0.18	0.00	0.26	2.69	0.31	0.07
		8.00	220.00	4.30	3.00	0.35	4.17	0.12	0.00	0.18	2.69	0.35	0.11
		12.00	220.00	4.30	3.00	0.35	4.12	0.02	0.00	0.02	2.67	0.35	0.07
		24.00	225.00	4.30	3.00	0.35	4.14	0.00	0.00	0.00	2.77	0.37	0.30
	Bagasse	8.00	180.00	4.69	2.74	0.17	1.71	0.96	0.04	0.64	1.80	0.07	0.60
		12.00	190.00	4.69	2.74	0.17	3.77	0.42	0.00	0.72	2.21	0.11	0.37
		20.00	200.00	4.69	2.74	0.17	4.48	0.19	0.00	0.33	2.44	0.11	0.14
		16.00	210.00	4.69	2.74	0.17	4.62	0.00	0.00	0.00	2.35	0.12	0.11
		12.00	220.00	4.69	2.74	0.17	4.55	0.00	0.00	0.00	2.32	0.11	0.10
		22.00	223.00	4.69	2.74	0.17	4.30	0.00	0.00	0.00	2.00	0.12	0.13

			untreated			pretreated solids			hydrolyzate			SSF residue
	time (min)	Temp (oC)	glucan	XMG	arabinan	glucan	XMG	arabinan	glucan	XMG	arabinan	glucan
Figure 3 and 4. Poplar	8.00	180.00	4.70	2.00	0.12	4.07	0.67	0.00	0.39	1.24	0.14	0.46
	16.00	180.00	4.70	2.00	0.12	4.20	0.38	0.00	0.37	1.31	0.14	0.43
	8.00	200.00	4.72	2.01	0.12	4.29	0.32	0.00	0.42	1.59	0.12	0.30
	16.00	195.00	4.99	2.12	0.13	4.90	0.30	0.00	0.18	1.73	0.14	0.23
	12.00	200.00	4.74	2.02	0.12	4.47	0.23	0.00	0.43	1.66	0.14	0.18
	12.00	200.00	4.72	2.01	0.12	4.04	0.22	0.00	0.41	1.70	0.13	0.18
	12.00	200.00	4.74	2.02	0.12	4.54	0.21	0.00	0.41	1.69	0.13	0.18
	16.00	200.00	4.70	2.00	0.12	4.13	0.26	0.00	0.41	1.66	0.14	0.11
	20.00	200.00	4.80	2.04	0.12	4.20	0.18	0.00	0.39	1.72	0.14	0.17
	16.00	205.00	4.99	2.12	0.13	4.70	0.19	0.00	0.22	1.80	0.13	0.22
	24.00	200.00	4.99	2.12	0.13	4.86	0.00	0.00	0.24	1.87	0.11	0.15
	28.00	200.00	5.00	2.13	0.13	4.94	0.20	0.00	0.20	1.97	0.11	0.26
	8.00	220.00	4.70	2.00	0.12	4.05	0.18	0.00	0.43	1.63	0.12	0.19
	16.00	210.00	4.83	2.06	0.12	4.79	0.13	0.00	0.42	1.94	0.12	0.14
	20.00	210.00	4.70	2.00	0.12	4.07	0.10	0.00	0.45	1.80	0.13	0.13
	24.00	210.00	5.03	2.14	0.13	4.81	0.00	0.00	0.24	1.87	0.09	0.17
	16.00	220.00	4.70	2.00	0.12	4.39	0.14	0.00	0.44	1.79	0.13	0.22
	20.00	220.00	4.99	2.12	0.13	4.83	0.00	0.00	0.22	1.84	0.10	0.23

		Time (hours)	Initial glucan (g)	Final glucan (g)
Figure5. SSF	FT pretreated poplar with ball milling	24.00	0.25	0.15
		48.00	0.25	0.09
		96.00	0.25	0.03
	FT pretreated poplar, 10 FPU/g glucan	24.00	0.25	0.15
		48.00	0.25	0.10
		96.00	0.25	0.05
	Avicel	24.00	0.25	0.16
		48.00	0.25	0.11
		96.00	0.25	0.07
	FT pretreated poplar, 5 FPU/g glucan	24.00	0.25	0.20
		48.00	0.25	0.16
		96.00	0.25	0.10
